# 立体定向放疗——Ⅰ期可手术非小细胞肺癌患者的更优选择？

**DOI:** 10.3779/j.issn.1009-3419.2015.06.01

**Published:** 2015-06-20

**Authors:** 冬梅 袁, 勇 宋

**Affiliations:** 210002 南京，南京军区南京总医院呼吸内科，南京大学医学院临床学院 Department of Respiratory Medicine, Jinling Hospital, Medical School of Nanjing University, Nanjing 210002, China

肺癌是发生率及死亡率最高的恶性肿瘤之一^[1]^，而肺叶切除是能够耐受手术的早期非小细胞肺癌（non-small cell lung cancer, NSCLC）患者治疗的金标准^[2]^。然而部分早期NSCLC患者因高龄、心肺功能储备不足或可能存在的手术并发症未能进行手术治疗^[3]^。

立体定向放疗（stereotactic ablative radiotherapy, SABR）利用计算机技术，在三维方向上与肿瘤靶区高度一致，在肿瘤靶区受到高剂量照射的同时最大限度地保护周围正常组织。与传统放疗相比，SABR能够延长患者生存，并减少放疗并发症的发生率^[4]^。但是SABR能否取代手术治疗，成为早期NSCLC患者的最优选择，这一问题尚存在争议。关于SABR与手术治疗的比较，目前已启动3项随机对照临床研究：STARS研究（NCT00840749）、ROSEL研究（NCT00687986）及ACOSOG Z4099研究（NCT01336894），但3项研究均因患者入组较慢而提前终止。Chang等^[5]^将STARS及ROSEL两项研究的数据进行汇集分析，结果发表在*The Lancet Oncology*，共纳入58例早期可手术切除的NSCLC患者（T1-2aN0M0）（31例患者行SABR治疗，27例行手术治疗），行SABR治疗者3年总生存（overall survival, OS）率为95%，而手术治疗者为79%[风险比（hazard ratio, HR）=0.14，95%CI: 0.017-1.190，*P*=0.037]。3年无复发生存（recurrence-free survival, RFS）率分别为86%及80%（HR=0.69, 95%CI: 0.21-2.29, *P*=0.54）。接受SABR治疗的患者中，3例（10%）患者出现3级治疗相关副作用，无4级治疗相关副作用或死亡患者。接受手术治疗患者中，1例（4%）患者死于手术并发症，12例（44%）患者出现3级-4级治疗相关副作用。该研究结果似乎更加推崇在早期NSCLC患者中进行SABR：延长了患者的OS，同时降低了治疗相关副作用的发生率。但是，该研究纳入病例数较少，统计效能较低，仍有一些问题需进一步探讨。

## 1 SABR *vs* 手术治疗，延长Ⅰ期NSCLC患者OS？

Chang等^[5]^的研究指出SABR在3年生存率、1年生存率方面均优于手术治疗，二者差异有统计学意义。当将STARS研究与ROSEL研究分别分析时，STARS结果与本研究一致，ROSEL研究中却未能得到证实（*P*=0.78）。我们认为作者应将ACOSOG Z4099纳入本研究，增加样本量，增加研究可信度，ACOSOG Z4099研究与另外两项研究相比，入组条件并无不同^[6]^。检索文献发现，Zhang等^[7]^对有关早期NSCLC患者SABR及手术治疗比较的队列研究或病例对照研究进行了荟萃分析，6项研究，864例患者，432例行SABR治疗，432例行手术治疗，总生存HR=1.82，95%CI:1.38-2.40，*P* < 0.001，与Chang等^[5]^的研究结果截然相反。两项研究结果不同的原因可能为：①Chang等^[5]^的研究为前瞻性的随机对照研究，研究样本量为58例，均为Ⅰ期NSCLC患者，而Zhang等^[7]^的荟萃分析纳入了队列研究或病例对照研究，共864例患者，为早期NSCLC患者；②Chang等^[5]^的研究纳入了较多的高龄患者，手术组中位年龄67岁，年龄最高达85岁，而高龄患者肺叶切除围手术期并发症发生率及死亡率均较高^[8]^，术后心肺功能代偿能力较差，复发后进行后续治疗的可能性较低。因此，较多高龄患者的入组有可能成为该研究中手术组患者生存较差的重要原因；③影响OS的因素本身较多，除了研究所关注的手术治疗（手术术式选择、手术相关并发症）或SABR（治疗剂量、次数、靶区划定）之外，肿瘤复发后的治疗（如化疗、靶向治疗、再次手术或放射治疗）同样重要；④在Zhang等^[7]^的荟萃分析纳入的回顾性研究中，更多的不能耐受手术的早期患者（如肺功能较差，或体质状况较差的患者）可能接受SABR的治疗，而这部分患者本身的生存可能就差于能够耐受手术的患者。因此，我们认为对于Chang等^[5]^研究中SABR延长早期NSCLC患者OS的结论尚需进一步斟酌。

## 2 SABR *vs* 手术治疗，相似的疾病控制率？

在疾病控制方面，Chang等^[5]^研究发现，SABR与手术相比，两组在无局部病灶复发率（96% *vs* 100%, *P*=0.44）、无局部淋巴结复发率（90% *vs* 96%, *P*=0.32）、无远处转移发生率（97% *vs* 91%, *P*=0.42）、3年无复发生存率（86% *vs* 80%, *P*=0.54）等方面均无统计学差异。Zhang等^[7]^的荟萃分析则指出二者在3年疾病控制率（HR=0.74, 95%CI: 0.46-1.20, *P*=0.22）及3年无病生存（HR=1.19, 95%CI: 0.71-1.99, *P*=0.51）方面差异无统计学意义。由此可见，SABR及手术治疗在疾病控制率方面的差异并不明显，而在局部病变的控制方面，手术治疗似乎稍有优势。既然手术组的局部控制及复发率方面稍优于SABR组，那么当对随机对照研究中纳入患者进行进一步筛选，并且延长随访时间，是不是患者的OS方面也会有不同的结果呢？

## 3 SABR *vs* 手术治疗，降低治疗相关副作用？

在治疗相关副作用方面，Chang等^[5]^指出，行SABR的患者3级-4级副作用发生的概率低于行手术治疗的患者（10% *vs* 44%）。在既往研究中，Verstegen等^[9]^同样发现SABR治疗的副作用发生率低于电视胸腔镜手术（video-assisted thoracoscopic surgery, VATS）（*P*=0.03）。Robinson等^[10]^认为在治疗相关死亡率（0% *vs* 1.5%）及治疗相关肺炎（7.7% *vs* 5.8%）发生率方面，两者之间差异无统计学意义。总体而言，上述研究在治疗相关副作用方面结果基本一致，即在早期NSCLC患者中，SABR发生治疗相关副作用或并发症的风险低于手术治疗。

## 4 小结

Chang等的研究对于Ⅰ期可手术NSCLC患者的治疗提出了一些新的观点，即SABR可能成为这类患者的新选择，而不仅仅是不能耐受手术的患者才考虑SARB治疗。但仅凭该项研究就可认为SABR取代手术治疗，成为早期NSCLC患者治疗的金标准吗？我们觉得为时尚早。我们对该研究的观点包括：①SABR与手术治疗相比，治疗相关并发症或副作用发生率较低；②在肿瘤控制方面，SABR并不劣于手术治疗；③对于高龄、肺功能较差，合并相关疾病的早期NSCLC患者，SABR无疑是较优选择，而对于可进行手术切除的早期NSCLC患者，我们仍更倾向于手术切除治疗，除了更为彻底地控制病变以外，手术切除病理带来的更准确的术后分期同样重要，SABR与手术治疗相比真的能够延长患者的OS吗？我们期待更多的随机对照临床研究给我们更为明确的答案。
